# A Facile Platform for One‐Step Generation of Uniform Microdroplets through Dehydration‐Driven Phase Separation in Microfluidics

**DOI:** 10.1002/smtd.202500387

**Published:** 2025-06-08

**Authors:** Ken Hirano, Mayu Shono, Akihisa Shioi, Kenichi Yoshikawa

**Affiliations:** ^1^ Health and Medical Research Institute National Institute of Industrial Science and Technology (AIST) Hayashi‐cho 2217‐14 Takamatsu Kagawa 761‐0395 Japan; ^2^ Department of Chemical Engineering and Materials Science Doshisha University Tatara Miyakodani 1–3 Kyotanabe Kyoto 610‐0321 Japan; ^3^ Komaba Institute for Science Graduate School of Arts and Sciences The University of Tokyo Komaba 3‐8‐1 Meguro Tokyo 153–8902 Japan; ^4^ Faculty of Life and Medical Sciences Doshisha University Tatara Miyakodani 1–3 Kyotanabe Kyoto 610‐0394 Japan

**Keywords:** aqueous two‐phase system, cell‐sized droplet, dehydration‐driven, liquid‐liquid phase separation, PDMS microfluidics, protocell generation, uniform droplet formation

## Abstract

Microdroplet generation with the desired size is essential in various fields; however, conventional methods require complex equipment and precise flow control, limiting their accessibility. To address this challenge, this research introduces a novel and straightforward method for one‐step generation of uniform, cell‐sized droplets using a simple microfluidic channel made of polydimethylsiloxane (PDMS). This approach exploits the inherent water‐absorption properties of PDMS to induce phase separation in a homogeneous aqueous two‐phase system comprising polyethylene glycol (PEG) and dextran (DEX). Injecting a homogeneous PEG/DEX mixture below the critical concentration for phase separation into the PDMS microchannel resulted in gradual dehydration, inducing microphase separation and generating linearly arranged DEX‐rich droplets within a PEG‐rich continuous phase. Time‐lapse observations revealed that this dehydration‐driven process is gradual and controlled, producing uniform droplet sizes. The key aspects of the observed phenomena are replicated through numerical simulations using a modified Cahn–Hilliard equation that accounts for the inherent water absorption characteristics of PDMS. Furthermore, the versatility of this method is demonstrated by the successful encapsulation of various materials, such as Escherichia coli, DNA, antibodies, and nanoparticles, within the droplets. This effective technique holds promise for a wide range of applications, such as drug delivery and artificial cell engineering.

## Graphical abstract

This study introduces a novel droplet generation method that exploits the intrinsic water absorption of polydimethylsiloxane (PDMS) microchannels. A homogeneous polyethylene glycol/dextran mixture is gradually dehydrated, leading to phase separation and the formation of uniform DEX‐rich droplets. This process spontaneously traps DNA, bypassing complex flow control systems, and enabling biomolecular encapsulation, highlighting its potential for drug delivery and artificial cells.

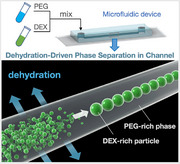

## 1 Introduction

Microscopic droplets formed via water‐in‐water (w/w) phase separation or aqueous two‐phase systems (ATPS) have attracted considerable interest in recent years. A century ago, Oparin proposed a hypothesis concerning the origin of life, suggesting that life on Earth originated from aqueous droplets, known as “coacervate drops,” composed of various organic materials including biomacromolecules.^[^ ^1^, ^2^ ^]^ Although initially overlooked owing to questions about microdroplet stability in the absence of a membrane barrier, this concept has gained renewed attention with the discovery that several membrane‐less organelles, such as nucleoli, Cajal bodies, P‐bodies, and stress granules, exist as liquid droplets within living cells. These structures are frequently interpreted in terms of liquid–liquid phase separation (LLPS) highlighting the relevance of coacervate‐like behavior in biological systems.^[^ ^3^, ^4^, ^5^, ^6^, ^7^, ^8^, ^9^ ^]^

In contrast, ATPS has long been recognized as a valuable method for the separation and purification of chemical substances, biomolecules, and cellular organelles. Since Albertsson's pioneering work in the 1950s, which established experimental conditions for selective partitioning between phases.^[^ ^10^ ^]^ Since then, ATPS with bulk phase separation has been actively applied in the separation of biological substances such as DNA, enzymes, monoclonal antibodies, viruses, and cells or organelles.^[^ ^11^ ^]^ In these studies, phase separation was induced using two polymers, such as polyethylene glycol (PEG) and dextran (DEX), or a polymer–salt system, with the two‐polymer systems being preferentially used for separation.

Over the past couple of decades, many biochemists and biophysicists have adopted the term LLPS more frequently than “coacervate” to interpret the structure and function of membraneless organelles.^[^ ^3^, ^11^, ^12^ ^]^ On some occasions, the term “coacervate” has been used to describe the physicochemical state of droplets caused by condensation or aggregation among macromolecules through attractive interactions, such as electrostatic interactions between cationic and anionic species, hydrogen bonding, and attractions among hydrophobic segments. It should be noted that the depletion effect, or crowding effect, due to incompatibility among polymer chains with different stiffnesses, also contributes significantly to the thermodynamics of segregation in two‐polymer systems, in addition to the attractive interactions among the same macromolecules.^[^ ^13^, ^14^, ^15^, ^16^, ^17^, ^18^ ^]^

Microfluidic platforms have emerged as promising tools for the preparation of aqueous droplets with desired sizes and compositions using ATPS.^[^ ^12^, ^19^, ^20^, ^21^, ^22^, ^23^, ^24^, ^25^, ^26^, ^27^ ^]^ In many studies, conventional microfluidic techniques are used to mix binary liquid microflows whose compositions are adjusted to undergo phase separation under stationary, non‐mixing conditions. In other words, many current studies on droplet formation via ATPS have adopted strategies similar to those used for oil–water systems.^[^ ^12^, ^19^, ^20^, ^21^, ^22^, ^23^, ^24^, ^25^ ^]^ The droplet formation process in these systems is highly dependent on shear forces generated by commonly used geometries such as T‐junctions and flow‐focusing devices. These techniques leverage high‐velocity fluid flows and rapid changes in channel geometry to induce shear and produce droplets. Although these shear‐based methods can generate monodisperse droplets, they typically require delicate control over flow rate, viscosity, and channel dimensions to achieve the desired droplet size and uniformity. Conventional techniques that rely on shear forces are almost incapable of producing multiple droplets simultaneously in a single step because they typically involve a sequential process of forming individual droplets. These limitations introduce additional complexity, reduce flexibility, and hinder the scalability of conventional droplet generation approaches in microfluidic applications.

Recently, we found that cell‐sized droplets entrapping DNA and F‐actin were generated spontaneously through simple mechanical mixing of aqueous solutions of binary polymers (PEG/DEX) together with DNA and F‐actin.^[^ ^28^ ^]^ Interestingly, with the addition of phospholipid suspension to the PEG/DEX binary solution containing DNA, it has been revealed that cell‐sized liposomes containing DNA molecules stabilized by phospholipid membranes emerge in a self‐organized manner.^[^ ^29^ ^]^ We have also reported that uniform cell‐sized droplets, as well as microgels, can be generated autonomously using a simple experimental procedure: introducing a binary polymer (PEG/DEX) solution into a glass capillary, where the composition of the binary polymer is set to cause phase separation while standing still.^[^ ^30^, ^31^, ^32^ ^]^

As an extension of these studies, in the present article, we report that uniform cell‐sized droplets can be formed spontaneously in a linear microfluidic channel fabricated from polydimethylsiloxane (PDMS). This is achieved through a simple procedure wherein a homogeneous solution of a binary polymer (PEG/DEX) is introduced into the channel and then allowed to remain stationary inside the PDMS channel. Following this treatment, linearly arranged microdroplets emerge in a self‐organized manner. We discuss the droplet formation mechanism in terms of the intrinsic water‐absorbing properties of PDMS. This novel methodology offers significant benefits in terms of simplicity compared to current droplet formation techniques using microfluidic technology, which often require delicate experimental setups with multiple microflow lines containing different liquid solutions.

## 2 Experimental Results

**Figure 1:**
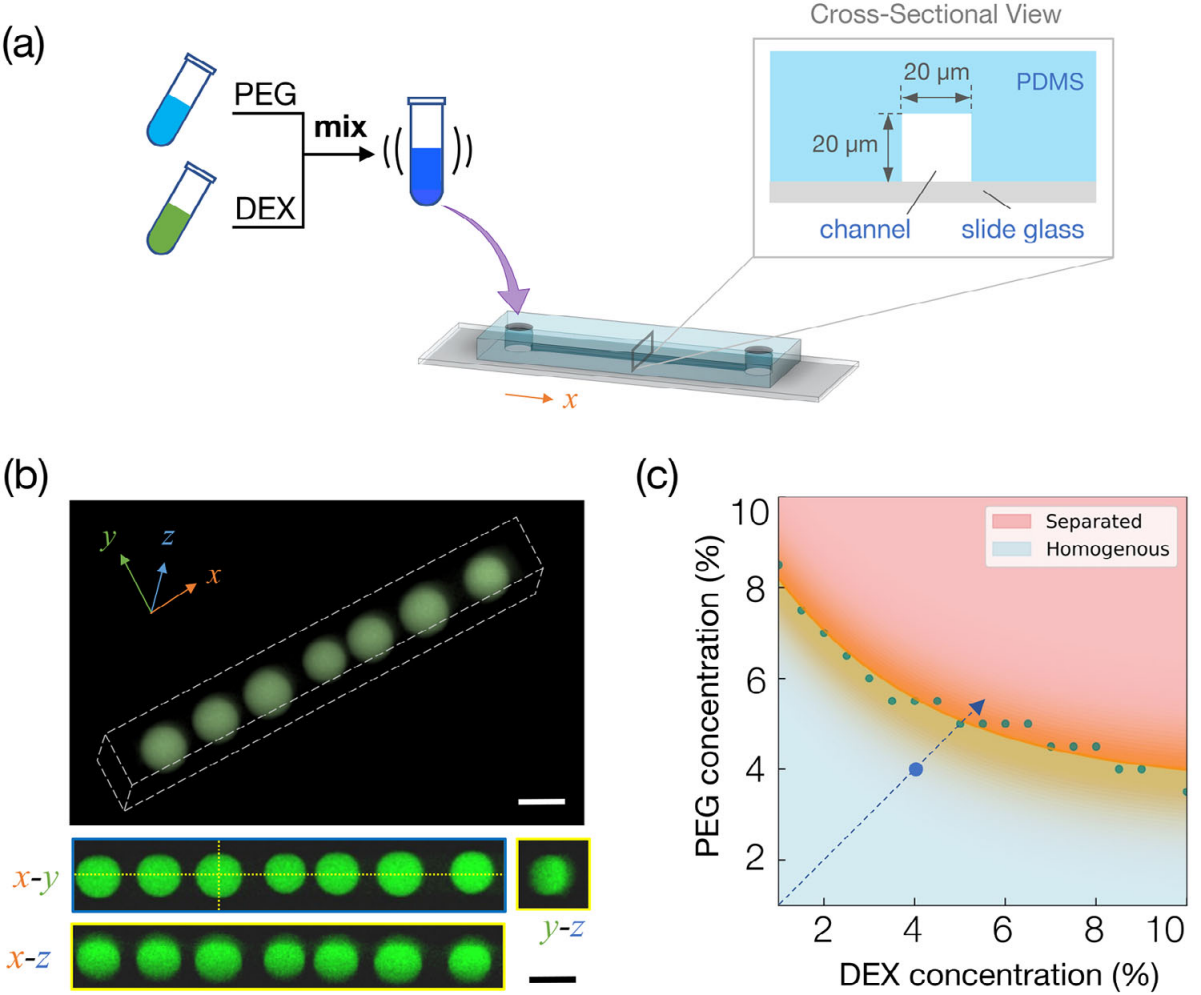
Self‐organization of linearly arranged micro w/w droplets along a microfluidic channel under the intrinsic effect of water absorption onto the channel material of PDMS. a) Scheme of the experimental setup. A homogenous aqueous solution with 4%:4% (w/v) PEG/DEX mixture was introduced into a linear microfluidic channel (inset: channel geometry, width, and height are both 20 µm and length is 30 mm. See Figure S1, Supporting Information for details). b) Confocal microscopic images of DEX‐rich droplets formed along the microfluidic channel. The top image shows a 3D reconstructed view of the 20 µm microfluidic channel after droplet formation. The dotted lines mark the microchannel boundaries. The bottom images show cross‐sections of the channel along the *x‐z* and *y‐z* planes (yellow dotted lines), revealing the uniform, spherical shape of the droplets (diameter ≈20 µm). Visualization was achieved by adding FITC‐dextran to the PEG/DEX solution in (b). Scale bars are 20 µm. c) Phase diagram of the PEG/DEX aqueous two‐phase system. The orange curve represents the binodal boundary based on previous literature (Tsumoto et al., 2015).^[^ ^35^ ^]^ Green data points were adapted from this reference. The blue circle indicates the initial composition (4% PEG, 4% DEX) used in our microfluidic experiments. The blue dashed line illustrates a hypothesized increase in polymer concentrations due to dehydration, eventually leading to phase separation.

**Figure** **1**a depicts the experimental setup, in which a homogeneous aqueous solution of PEG and DEX below the binodal line in the phase‐separation diagram was introduced into a linear microfluidic channel fabricated from PDMS. The channel had a uniform square cross‐sectional shape along its length (see Figure S1, Supporting Information for details). PDMS is inherently hydrophobic and highly porous, enabling it to gradually absorb water vapor from the aqueous PEG/DEX solution confined within the microchannel.^[^ ^33^, ^34^ ^]^ To facilitate bonding to a glass substrate and to allow aqueous solution loading, the PDMS surfaces were treated with oxygen plasma, which temporarily introduces polar functional groups and renders the inner channel walls hydrophilic. All experiments were performed within 6 h of plasma activation to maintain sufficient surface wettability. While plasma treatment affects only the surface, the bulk properties of PDMS remain unchanged or may even show enhanced water vapor transmission. For instance, Bian et al. reported a 60% increase in water vapor transmission following plasma activation, attributed to accelerated sorption at the activated hydrophilic surface.^[^ ^33^ ^]^ In our system, dehydration of the PEG/DEX solution occurs predominantly through vapor diffusion across the PDMS wall, rather than bulk liquid absorption. This process is governed by Fick's law and results in a gradual increase in local polymer concentrations, eventually leading to phase separation inside the PDMS microchannel within a few minutes under ambient conditions. Upon exceeding the critical concentration or crossing the binodal line of the phase diagram, the homogeneous PEG/DEX mixture undergoes phase separation. As shown in Figure 1b, fluorescence microscopy using fluorescein isothiocyanate (FITC)‐dextran to highlight the DEX‐rich phase effectively captured the phase transition from a homogeneous mixture to linearly arrayed droplets. Confocal microscopic observation was performed to confirm the size uniformity and spherical nature of the droplets with a 3D perspective. The confocal microscopy images in Figure 1b demonstrate that the droplets are not only uniformly arranged in a linear fashion along the microchannel but also exhibit a consistent spherical morphology throughout the channel. To provide further insight into the phase behavior of the PEG/DEX mixtures used in this study, Figure 1c presents an approximate phase diagram that delineates the boundary between homogeneous and phase‐separated regions. The binodal curve was adapted from our prior measurements,^[^ ^35^ ^]^ and the initial concentrations of 4%:4% are annotated on the plot. This representation confirms that the 4%:4% solution initially resides in the homogeneous regime and thus requires dehydration to trigger phase separation, whereas the 5%:5% solution lies above the binodal and undergoes spontaneous separation. This contrast underscores the significance of controlled dehydration in enabling uniform droplet formation from an initially single‐phase system.

**Figure 2:**
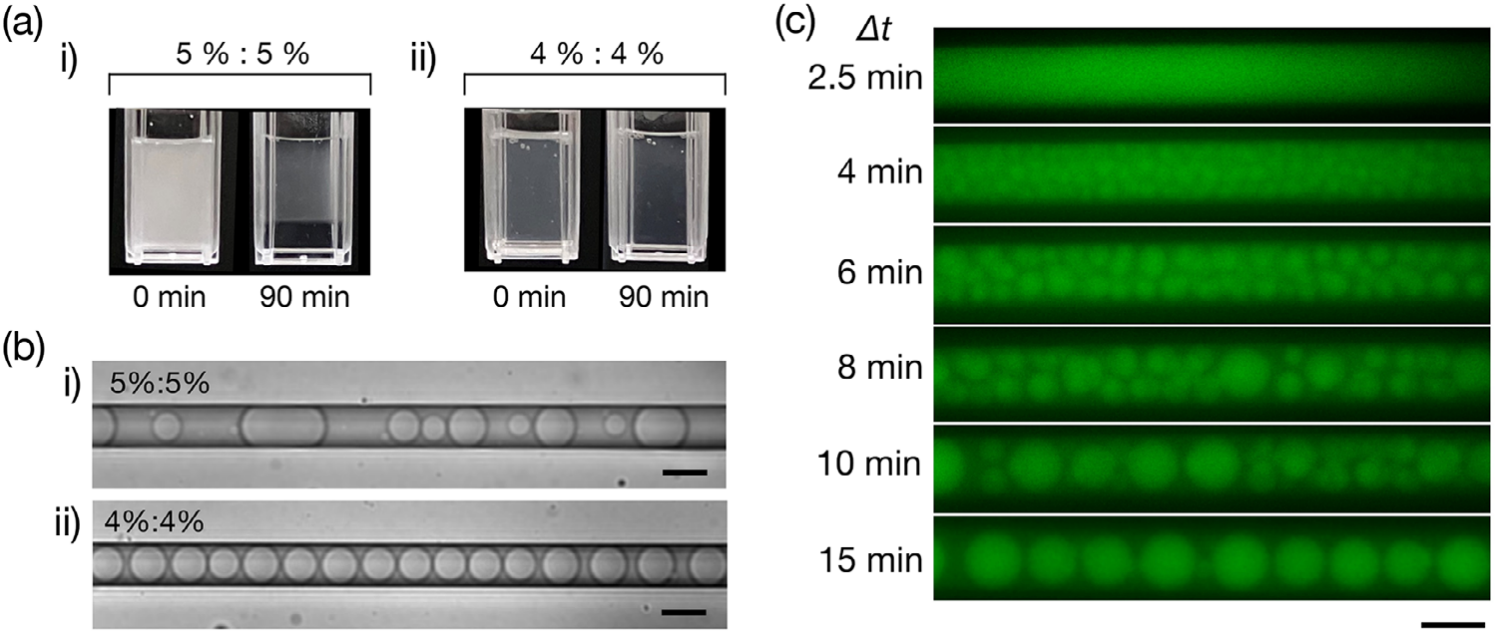
Microscopic observation of concentration‐dependent and spontaneous droplet formation. a) Phase behavior of PEG/DEX aqueous solution under stationary conditions after mechanical agitation. While a 5%:5% (w/v) PEG/DEX solution exhibits phase separation (i), no visible separation was observed in a 4%:4% (w/v) solution (ii). Each cuvette has a width of 1 cm. b) Microscopic images of spontaneously generated droplets. The bright‐field images demonstrated the outcome of introducing a 4%:4% (w/v) PEG/DEX mixture (i) and a 5%:5% (w/v) PEG/DEX mixture (ii), prepared with pure water. For the 4%:4% mixture, uniform‐sized DEX‐rich droplets (diameter ≈20 µm) are observed. The images were taken 15 min after the introduction of the mechanically mixed solution. c) Time‐lapse fluorescence microscopy images of a 4%:4% (w/v) PEG/DEX mixture under slow fluid flow (≈5 µm min^−1^) in the microfluidic channel at Δ*t* = 2.5, 4, 6, 8, 10, and 14 min after introducing the mixture. Droplet formation progresses over time, resulting in a single row of uniform‐sized droplets (diameter ≈ 20 µm). See Movie S1 (Supporting Information). Visualization was achieved by adding FITC‐dextran to the PEG/DEX solution. Scale bars are 20 µm. The presented images correspond to the middle portion of the microchannel.

**Figure** **2**a shows the difference in the macroscopic behavior between 5%:5% and 4%:4% (w/v) PEG/DEX solutions left standing for 0 and 90 min after mechanical mixing. The 5%:5% solution displays the formation of upper and lower phases after 90 min, whereas the 4%:4% solution remains in a transparent homogeneous state (indicating it is outside the binodal line in the phase diagram). In Figure 2b, the different behaviors of these polymer solutions inside the PDMS microchannel are shown, observed 15 min after the introduction of the mechanically mixed solutions. To minimize external humidity effects during long‐term observation, a thin layer of pure water was placed on the outer surface of the PDMS device immediately after droplet formation. This environmental stabilization step reduced further dehydration and allowed for consistent assessment of droplet morphology. We confirmed that these droplets were stable and maintained their shape over a period of several hours (Figure S2, Supporting Information). In other words, ripening phenomena common in the usual phase separations of polymer solutions^[^ ^36^, ^37^ ^]^ are inhibited inside the PDMS channels after the formation of the linearly aligned droplets. Neither fusion between neighboring droplets nor the growth of certain droplets at the expense of others occurs over several hours. The formation of such stable droplets alongside a narrow quasi‐1D system was reported in our previous studies, where we discussed the mechanism in terms of the quasi‐1D confinement effect.^[^ ^30^, ^31^, ^32^ ^]^ Time‐dependent changes in the 4%:4% solution are shown in Figure 2c and Movie S1 (Supporting Information), depicting the ripening and coarsening of the generated small droplets in the initial stage (less than 10 min) and the formation of an array of stable, similarly sized droplets at 15 min. It is important to note that the droplets observed here are generated from a 4%:4% (w/v) PEG/DEX solution, which is initially homogeneous and below the binodal line in the phase diagram. This highlights a key advantage of the PDMS‐based system, where controlled dehydration induces microphase separation within the channel itself; an effect not achievable in glass‐based systems without pre‐phase‐separated inputs, as will be further discussed. In the later part of the present paper, we will discuss the mechanisms behind the formation of a 1D array of stable, similarly sized droplets based on the numerical results of theoretical modeling of phase separation under quasi‐1D confinement.

**Figure 3:**
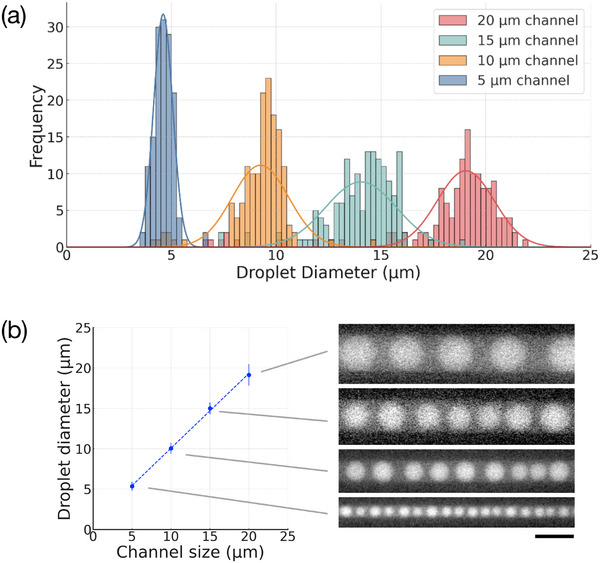
Control of droplet diameter by channel size. a) Histograms of droplet diameters generated in 5, 10, 15, and 20 µm‐wide PDMS microchannels, along with corresponding Gaussian fits. Each condition adopted a tailored PEG/DEX concentration (1%, 2%, 3%, and 4%, respectively) to optimize phase separation timing. b) Relationship between channel size and average droplet diameter. Error bars indicate standard deviations (*n* = 140 droplets per condition). Representative fluorescence images of droplets in each channel are shown on the right. The scale bar is 20 µm.

To further evaluate the controllability of droplet size, we systematically varied the width of the PDMS microchannels (**Figure** **3**). We tailored the initial polymer concentrations according to the dehydration kinetics: 1%:1% (w/v) PEG/DEX for 5 µm channels (≈3 min), 2%:2% for 10 µm channels (≈7 min), 3%:3% for 15 µm channels (≈8 min), and 4%:4% for 20 µm channels (≈10 min). Further details regarding the selection of these concentrations and the corresponding dehydration kinetics are provided in the Supporting Information (Section S2 and Figure S6, Supporting Information). The resulting average droplet diameters, determined by fluorescence microscopy, were ≈4.6, 9.3, 14.1, and 18.3 µm, respectively, closely matching the channel dimensions. The corresponding coefficients of variation (CV) were 9.8%, 14.6%, 12.1%, and 16.3%, respectively, demonstrating high size uniformity across all conditions (Figure 3a). The relationship between channel width and droplet diameter exhibited excellent linearity (Figure 3b), validating the scalability of the dehydration‐driven phase separation mechanism. Notably, in the 20 µm channel condition, a minor fraction of satellite droplets (<14 µm) was detected. Excluding these satellites yielded a recalculated average diameter of 19.0 µm and an improved CV of 7.16%, highlighting the intrinsic monodispersity of the main droplet population.

**Figure 4:**
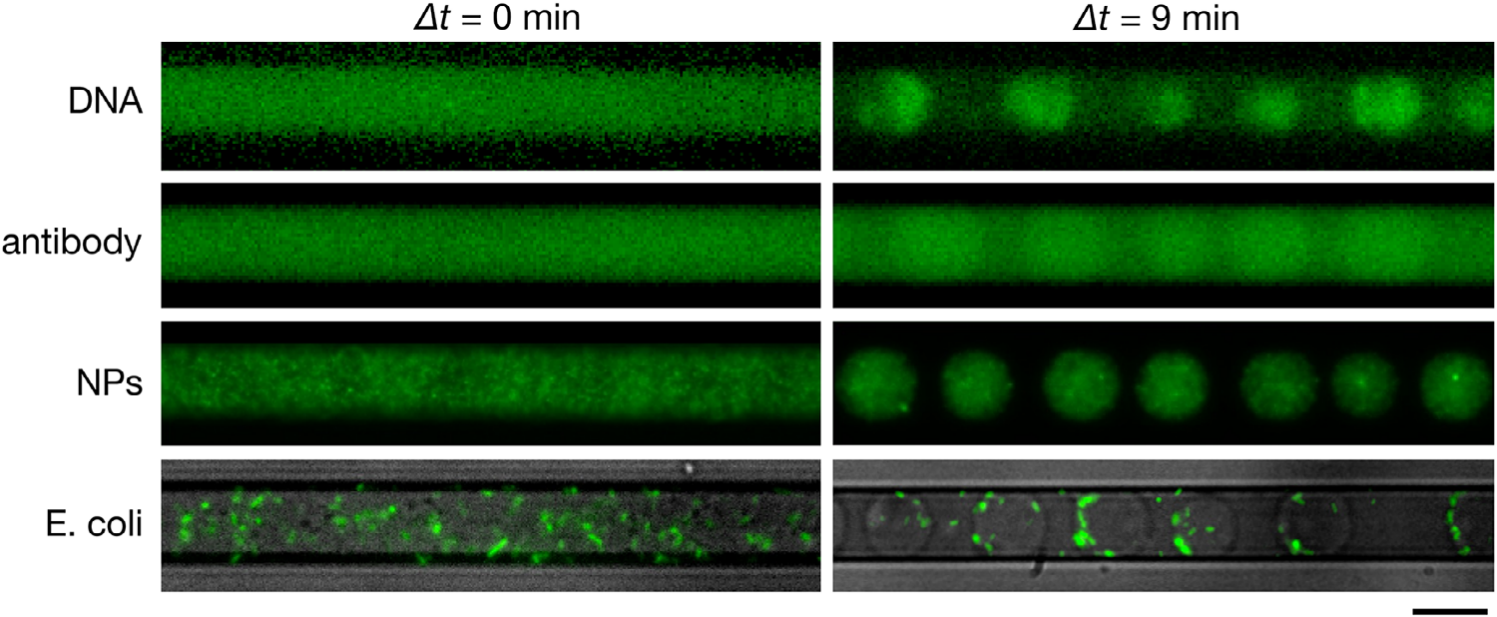
Encapsulation of various substances within dextran‐rich droplets. Fluorescence microscopy images of DEX‐rich droplets formed in a microfluidic channel from a 4%:4% (w/v) PEG/DEX mixture, prepared with 1 × PBS, entrapping T4 phage DNA (166 kbp, ≈56 µm) stained with YOYO‐1, anti‐mouse IgG antibody labeled with Alexa Fluor 488, 200 nm carboxylate‐modified fluorescent polystyrene beads (nanoparticles, NPs), and Escherichia coli (E. coli) expressed GFP (bright‐field and fluorescence images merged). Images in the left and right columns show the initial state immediately after the introduction of the PEG/DEX mixture and the separated state of droplets 9 min after the introduction. The scale bar is 20 µm.

**Figure** **4** demonstrates the successful encapsulation of a variety of materials—including Escherichia coli (E. coli) expressing green fluorescent protein (GFP), T4 phage DNA, an anti‐mouse IgG antibody, and 200 nm carboxylate‐modified fluorescent polystyrene beads—within the dextran‐rich droplets formed from a 4%:4% PEG/DEX mixture. In a related study, we reported that PEG‐rich droplets spontaneously entrapped mammalian cells, including erythrocytes and epithelial cells, by utilizing the ATPS with PEG and DEX.^[^ ^38^, ^39^ ^]^ These findings demonstrate the versatility of this method for encapsulating a diverse range of materials, including biomolecules, cells, and synthetic particles. This capability is relevant to the fields of artificial cell engineering and bottom‐up synthetic biology, as the cell‐sized droplets demonstrated in this study can serve as a potential platform for constructing artificial cells by encapsulating biomacromolecules and organelles.^[^ ^36^ ^]^ The dextran‐rich phase provides a cytosol‐like environment and a confined space for biomacromolecules and organellae to reside. This versatility offers a range of potential applications in drug delivery, cell‐based therapies, and materials science, as well as opportunities for advancing research on the origin of life and models of primitive cells.

## 3 Numerical Modeling

Next, we performed a numerical simulation to clarify the underlying mechanism for the formation of a regular array of similarly sized micro w/w droplets in the PDMS channel. For simplicity, we adopted a 2D modeling approach to evaluate the effect of the microchannels on the phase‐separation growth process. To interpret the observed phase separation phenomena, we used the Cahn–Hilliard equation:^[^ ^40^, ^41^, ^42^, ^43^, ^44^, ^45^, ^46^ ^]^

(1) $$\frac{\partial \eta }{\partial t}=\nabla \left(M_{c}\nabla \frac{\delta F}{\delta \eta }\right)$$

where *F* is the free energy, *t* is the time, and *M*_c_ =  (*D*_0_/*RT*) is the diffusivity parameter (*D*_0_ is the diffusion constant, *R* is the universal gas constant, and *T* is the absolute temperature). The relative ratio of DEX to the total PEG+DEX solution is represented as the parameter *η*, where *η*  =  [0,  1]. *η*  =  0.0 corresponds to the state of 100% PEG without DEX. The free energy *F* was approximated by considering the symmetry of the phase separation or first‐order phase transition as follows:

(2) $$F=\int \left(L\eta \left(1-\eta \right)+\frac{\alpha }{2}{\left|\nabla \eta \right|}^{2}\right)dV$$

where *L*, *α*, and *dV* denote the parameters representing the interaction and interfacial energy between PEG and DEX phases and differential volume, respectively. The first term, multiplied by *L* is the interaction energy, which was adopted for simplicity and generality. The second term multiplied by *α* represents the energy cost due to the spatial gradient of *η*.

When the PDMS channel was in a dry state before the incorporation of the polymer aqueous solution, its inherent hydrophilicity/permeability caused it to gradually absorb water from the ATPS solution within the channel. As shown in Figure 2c, this gradual dehydration process led to an increase in the concentrations of both PEG and DEX over several minutes. To minimize the number of variables and adjustable parameters in the numerical calculation, we approximated the interaction parameter *L* to evaluate the effect of water absorption as a function of the increase in polymer concentration. The interaction parameter *L* is given as follows to interpret the increasing dissociation effect between PEG and DEX over time caused by water absorption:

(3) $$L=L_{0}+a*\left(1-10^{-b*t}\right)$$

where *L*_0_ denotes the initial condition of the interaction parameter. Parameters *a* and *b* are constants. Here, we show the results of the simulation by taking $L_{0}=7300,\;a=10000,\mathrm{and}\;b=5.0\;\times \;10^{-3}$ s^−1^. For simplicity, we performed the simulation under a no‐flux boundary at the surface with respect to the variable *η*, adopting the boundary condition that the surface exhibits affinity to PEG.

**Figure 5:**
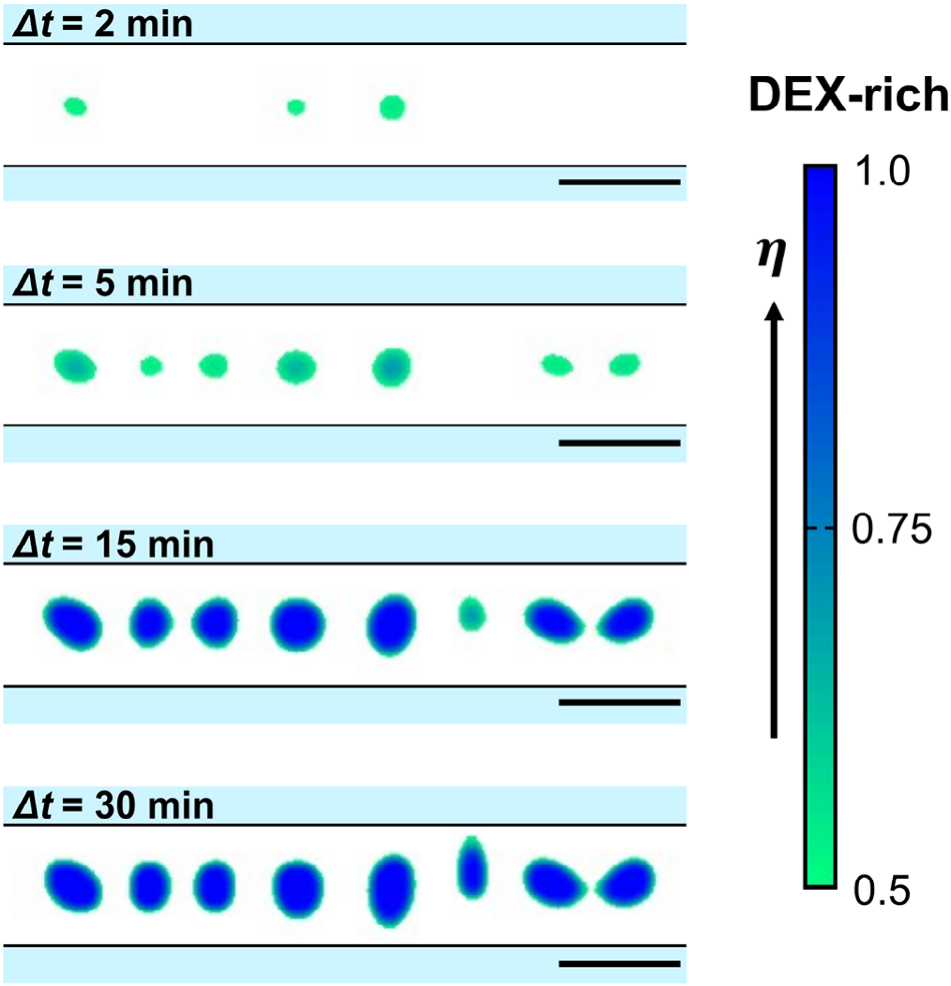
Numerical results regarding the time evolution of the phase separation of a PEG/DEX mixture within a microchannel (Δ*t*  =  2,  5,  15 and 30 min). The numerical calculation was performed with the model equations (Equations (1), (2), (3)). It seems that microphase separation occurs during the process of pouring it through the channel in the experiments, so the time parameter of numerical calculation was set to Δ*t*  =  *t* − 800 s, where *t*  =  0 corrsponds to the starting time of the simulation. The color map represents the ratio of PEG and DEX (*η*  =  [0.5,  1]). *η*  =  1.0 corresponds to 100% DEX without PEG. Scale bars are 20 µm.

**Figure** **5** shows the time‐dependent change in w/w segregation by using the time parameter, Δ*t*. We conducted the simulation by adopting perfect homogeneity (*η*  =  0.38) for the initial condition. (For a detailed consideration of Δ*t*, see the subhead Numerical Details in Experimental Section). The color map represents the ratio of PEG and DEX (*η*  =  [0.5,  1]). *η*  =  0.0 and 1.0 corresponds to 100% PEG and 100% DEX, respectively. Initially, small DEX‐rich droplets are generated through phase separation. These droplets tend to grow inside the channel, and then almost uniformly sized droplets spontaneously arrange themselves along the long axis in a stationary manner maintained for 30 min. The generation and maintenance of the uniform‐sized droplets can be attributed to the 1D of the system and the boundary attraction of the PEG phase. The process functions to generate almost uniform‐sized droplets, preventing the droplets from expanding beyond the diameter of the channel. In the experiments shown in Figure 2c, several small droplets appear in the initial stage of 4–8 min, which is somewhat different from the results of the numerical simulation in Figure 5. This discrepancy is attributable to the rough approximation in the calculation of the partial differential equation, where the grid spacing was taken as 1.0 µm to simplify the calculation. It is expected that the appearance of a number of smaller droplets (less than 1 µm) in the initial stage of phase segregation would be achieved by taking much smaller grid spacing, though this would require a larger amount of computation. Thus, these numerical results reproduced the essential behavior of the phase separation of the PEG/DEX solution in the microchannel observed in the present study.

## 4 Discussion

This study demonstrates a novel approach for generating uniform microdroplets through dehydration‐driven phase separation in PDMS‐based microfluidics. This method uniquely leverages the intrinsic water‐absorption properties of PDMS to induce phase separation in an aqueous PEG/DEX system, leading to the formation of dextran‐rich droplets within a PEG‐rich continuous phase. Unlike conventional microfluidic droplet generation methods that rely on shear force and external actuation,^[^ ^12^, ^19^, ^20^, ^21^, ^22^, ^23^, ^24^, ^25^ ^]^ this process occurs in a self‐organized manner without requiring precise flow control. The gradual dehydration of the PEG/DEX mixture within the microchannel results in a controlled increase in polymer concentration throughout the PDMS channel, allowing phase separation to occur in a highly uniform and reproducible manner. The uniformity of the generated droplets suggests that the rate of dehydration plays a crucial role in regulating phase separation dynamics. Compared to conventional phase separation methods, which often result in polydisperse droplet sizes due to rapid nucleation and coalescence, the gradual nature of this process helps maintain a low supersaturation level, leading to controlled nucleation and minimal size variation. The inclusion of the PEG/DEX phase diagram (Figure 1c) clarifies the distinct phase behaviors of the initial solution compositions used in this study. Notably, the 4%:4% (w/v) mixture lies below the binodal line, confirming its homogeneous nature at the outset. In contrast, the 5%:5% condition resides just within the two‐phase region, where spontaneous phase separation leads to rapid and heterogeneous droplet formation. This supports the interpretation that gradual dehydration allows the system to cross the binodal in a controlled manner, promoting uniform nucleation and monodispersity. Time‐lapse fluorescence imaging revealed that the transition from a homogeneous solution to a well‐ordered array of microdroplets occurs in distinct stages: beginning with the appearance of nano‐sized segregation, followed by their coalescence into stable, evenly spaced droplets. This behavior indicates that the dehydration process governs not only the initiation of phase separation but also the spatial arrangement of the resulting droplets within the confined microchannel.

An intriguing finding of this study is the stability of the droplets formed over extended periods. Unlike bulk‐phase ATPS, where droplet growth and Ostwald ripening typically lead to coarsening and eventual phase separation into distinct macroscopic layers, the microfluidic environment appears to suppress these effects. The confinement within the PDMS microchannel likely contributes to the stabilization of the droplets, preventing fusion and disproportionation. This phenomenon may be attributed to the quasi‐1D nature of the microchannel, which limits droplet mobility and inhibits large‐scale coalescence. The numerical simulations performed in this study further support this interpretation, demonstrating that a gradual increase in polymer concentration can drive phase separation in a controlled manner while maintaining a stable droplet array over time. To quantitatively assess droplet stability over time, we analyzed the diameter distribution at 15 min and at 2 h post‐formation (Figure S2, Supporting Information). At 15 min, droplets exhibited a narrow size distribution, while at 2 h, despite a few larger, ellipsoidal droplets forming due to partial fusion between adjacent droplets, the overall distribution remained comparable. Importantly, no systematic shrinkage of droplets was observed. These results support the conclusion that the generated microdroplets maintain their morphology for at least 2 h under ambient conditions. This stability is further supported by a simple environmental control: although PDMS remains permeable to water vapor, applying a water layer over the device after droplet formation effectively suppresses further dehydration by creating a near‐saturated external humidity environment. This approach suppresses the impact of seasonal or daily temperature/humidity fluctuations in usual experimental rooms without rigid air conditioning and enhances the reproducibility of droplet stability. The effectiveness of this strategy is evidenced by the consistent droplet morphology observed over 2 h (Figure S3, Supporting Information).

The key advantages of this method include its biocompatibility and simplicity. Unlike traditional oil‐in‐water or water‐in‐oil droplet generation techniques, which often require surfactants or external phase‐stabilizing agents, this approach uses exclusively aqueous components. Moreover, given that PDMS absorption functions via water vapor transmission,^[^ ^33^ ^]^ solute loss is effectively minimized. The elimination of surfactants is particularly advantageous for applications involving biological materials, as surfactants can introduce cytotoxic effects or interfere with biomolecular interactions. The ability to encapsulate biomolecules such as DNA, proteins, and bacterial cells without additional stabilizing agents highlights the potential of this system for biomedical applications, including controlled drug delivery, artificial cell engineering, and biochemical reaction compartmentalization.

Although the present study focused on PEG/DEX as a model ATPS, this approach may be extended to other polymer systems with tunable phase separation properties. The adaptability of the dehydration‐driven mechanism suggests that similar methodologies can be applied to other biopolymer‐based systems, potentially expanding the scope of their applications in biosensing, soft matter physics, and materials synthesis. Additionally, further modifications to the PDMS microchannel properties, such as surface functionalization or controlled humidity conditions, could enable finer control over the droplet formation process, allowing for more precise manipulation of droplet size and stability. This suggests potential applications in the fabrication of biocompatible DDS (drug delivery system) particles and other bio‐applicable microparticles. Additionally, the approach has the potential to be employed in uniform microreactors for enzymatic reactions, where, due to the nature of the water‐in‐water system, biochemical reactions could facilitate the transfer of substances between the phases, mimicking metabolic processes in cells. Moreover, it could serve as a template for microsphere synthesis using water‐soluble materials, further expanding its utility.

Microfluidic devices facilitate the parallelization of microchannels, indicating the potential for high‐throughput droplet production. Furthermore, based on the observed droplet densities (≈10 droplets per 250 µm channel length), we estimate that ≈400 droplets can be generated per centimeter in a single microchannel. With a channel pitch of 150 µm, scalable parallelization up to 100 channels per centimeter is feasible, corresponding to a production of ≈4 0000 droplets per centimeter width. These results suggest that the dehydration‐driven approach is not only robust but also scalable for high‐throughput applications. To explore the practical feasibility of channel parallelization, we experimentally evaluated the effect of channel spacing on droplet formation dynamics. Although PDMS enables scalable microfluidic architectures, we observed that excessive integration of adjacent channels may lead to inter‐channel competition for vapor diffusion (Figure S4, Supporting Information). When the spacing between channels was less than 150 µm, droplets formed later in central channels compared to edge channels, likely due to the local saturation of the PDMS matrix. However, a spacing of 150 µm mitigated this effect, enabling uniform and simultaneous phase separation. This finding highlights a practical constraint in designing high‐throughput systems based on dehydration‐driven ATPS.

To further investigate encapsulation behavior, we conducted fluorescence line profiling for DNA, nanoparticles (NPs), and antibodies, as shown in Figure S5 (Supporting Information). Strong and well‐localized fluorescence signals were observed for DNA and NPs within the dextran‐rich droplets, indicating effective partitioning. Antibodies, on the other hand, exhibited weaker fluorescence intensity across the droplet region, yet with no significant heterogeneity between droplets, suggesting relatively low but uniform encapsulation. To supplement these qualitative observations, we manually counted 80 droplets per sample type using representative fluorescence images. The measured encapsulation efficiencies were 100% for DNA, 100% for NPs, and 96.6% for E. coli. The slightly reduced efficiency for E. coli is attributed to partial cell adhesion to the PDMS wall. These results support the robustness of the dehydration‐driven method under the present experimental conditions. Regarding E. coli, the particulate fluorescence signal appeared as discrete dots, making line profile analysis impractical. Nonetheless, their presence within droplets was clearly confirmed by fluorescence microscopy (Figure 4), consistent with previous work.^[^ ^30^ ^]^ For antibodies, we did not perform droplet‐by‐droplet quantification because the weak and diffuse fluorescence signal under PBS conditions hindered reliable droplet‐level discrimination. Instead, antibody encapsulation was qualitatively evaluated using line profiles shown in Figure S5 (Supporting Information). Importantly, encapsulation efficiency is known to be concentration‐dependent. At lower concentrations of encapsulant molecules or particles, stochastic exclusion events may become more frequent, leading to reduced efficiency. In this study, relatively high concentrations were used to ensure consistent incorporation. In addition to molecular properties such as size and charge, the higher‐order structure of biomolecules has been reported to significantly affect their partitioning behavior within aqueous two‐phase systems, as demonstrated by Nishio et al.^[^ ^47^ ^]^ The behavior of proteins in ATPS systems can also be affected by pH, ionic strength, and the molecular characteristics of both the polymers and the proteins themselves. Asenjo and Andrews reported that protein partitioning between PEG‐rich and DEX‐rich phases varies significantly depending on the charge, hydrophobicity, and conformation of the target protein.^[^ ^48^ ^]^ In our current study, antibody encapsulation was conducted in standard PBS buffer without optimization, and further improvements may be achieved by modifying the buffer composition. These findings collectively suggest that the dehydration‐driven approach not only enables uniform droplet generation but also facilitates reliable encapsulation of various biochemical species, depending on tunable physical and chemical parameters.

In this study, we used PDMS microfluidic devices with a consistent thickness of ≈5 mm ± 1 mm, which enabled reproducible dehydration behavior under ambient laboratory conditions. Notably, PDMS membrane thickness has been reported to significantly influence water vapor transmission rates, especially in the thin‐film regime.^[^ ^33^ ^]^ Our selected thickness falls above this regime and provides a practical balance between permeability and mechanical robustness. To minimize experimental variability due to ambient humidity fluctuations, we implemented a humidity‐stabilization step by overlaying a thin water layer on the PDMS surface after droplet formation (Figure S3, Supporting Information). This strategy was introduced as a precautionary measure after we observed that, under our laboratory conditions in humid Japan, droplet stability over a period of at least 2 h, and occasionally slightly longer, could slightly vary depending on daily fluctuations in temperature and humidity. Minor size variation or occasional partial coalescence was observed in some instances. It has been confirmed that the presence of the water layer effectively suppressed such variability, although these effects were not systematically quantifiable, yet. Furthermore, this strategy is expected to improve reproducibility in laboratory environments with less humidity. PDMS remains the most commonly used elastomer for microfluidic prototyping owing to its optical clarity, gas permeability, and moldability.^[^ ^49^ ^]^ However, elastomer type and its hydration state can significantly influence vapor transport characteristics and dehydration kinetics. For broader applicability and industrial translation, a systematic investigation involving materials with varied water sorption and permeability properties—as well as diverse channel geometries and solution compositions—will be essential to optimize and generalize the proposed approach.

To complement our experimental observations, we considered a simple model based on diffusion‐limited dehydration governed by Fick's second law. As detailed in the Supporting Information (Section S2, Supporting Information), the characteristic time for reaching the critical concentration can be approximated by a quadratic or exponential dependence on channel size, consistent with the size dependence observed experimentally. This analysis highlights that both the initial polymer concentration (*C₀*) and the microchannel size significantly influence the timing of droplet formation, with lower *C₀* and larger channels leading to slower phase separation. Future research will focus on enhancing the dehydration performance of PDMS‐based devices to accommodate a broader range of initial polymer concentrations, thereby extending the applicability of the method to more diverse ATPS conditions. In parallel, we are currently exploring new strategies for spatial control and tunability of droplet morphology by implementing parallel channels filled with high‐osmotic‐pressure media adjacent to the PEG/DEX solution. These efforts aim to improve the uniformity and arrangement of droplets within the confined microchannel architecture.

Overall, these findings highlight the robustness and versatility of dehydration‐driven phase separation as a strategy for generating highly uniform microdroplets in a confined microfluidic environment. The combination of experimental observations and numerical modeling provides valuable insight into the underlying mechanisms governing this process, paving the way for future advancements in ATPS‐based droplet microfluidics.

## 5 Conclusion

This study presents a novel and efficient one‐step droplet generation method that harnesses the unique dehydration properties of PDMS for controlled water‐in‐water (w/w) phase separation in microfluidics. By leveraging the intrinsic water absorption capacity of PDMS, a homogeneous PEG/DEX mixture introduced into the microchannel undergoes gradual dehydration, resulting in an increase in polymer concentration and driving phase separation. This straightforward approach results in the formation of uniformly sized droplets, as evidenced by microscopic visualization. In our previous study, it was demonstrated that a linear arrangement of uniform droplets was generated by using a glass capillary through the procedure to introduce mechanically mixed PEG/DEX solution with the composition to cause phase separation (Shono, et al., Sci. Rep., 2021).^[^ ^30^ ^]^ In contrast, the present approach uniquely enables the formation of well‐aligned, monodisperse droplets starting from a fully homogeneous solution, highlighting the advantage of dehydration‐induced in situ phase separation within the PDMS microchannel. Significantly, this method obviates the need for intricate control over flow rates, viscosities, and channel geometry, which are typically required in conventional droplet generation techniques. The fundamental phenomena were reproduced by theoretical calculations based on the Cahn–Hilliard mechanism, which considers the inherent water‐absorption characteristics of PDMS. Furthermore, the versatility and potential of this method are further demonstrated by its ability to encapsulate a variety of substances in dextran‐rich droplets.

As an immediate extension of the present study, we are currently conducting experiments aimed at controlling the uniformity, morphology, and size of the aligned droplets by introducing parallel channels filled with high‐osmotic‐pressure media adjacent to the binary polymer solution. This innovative approach establishes a foundation for future research aimed at expanding its applicability to other polymer systems, including gelation processes (e.g., Shono et al., Small, 2023),^[^ ^31^ ^]^ and improving control over droplet properties. By refining the dehydration process and exploring functionalization strategies, this technique shows promise for advancing targeted drug delivery, biosensing, and artificial cell construction.

## 6 Experimental Section

### Materials

Polyethylene glycol PEG (*Mw* = 7300–9300 Da), DEX (*Mw* = 18 0000–21 0000 Da), and phosphate‐buffered saline PBS (10×, pH 7.4) were purchased from Fujifilm Wako Pure Chemical Corporation (Osaka, Japan). PDMS (SILPOT 184) was purchased from Dow Corning Toray Co., Ltd. (Tokyo, Japan). The T4 phage DNA (≈166 kb; T4 GT7 DNA) was purchased from Nippon Gene (Tokyo, Japan). Alexa Fluor 488 Goat Anti‐mouse IgG (H+L) (2 mg mL^−1^, *Ex*: 488 nm/*Em*: 519 nm), FluoSpheres carboxylate‐modified polystyrene beads (200 nm, 2% solid solution, yellow‐green fluorescent, *Ex*: 505 nm/*Em*: 515 nm), YOYO‐1 as a fluorescent dye were purchased from Thermo Fisher Scientific (Molecular Probes brand). A fluorescein isothiocyanate–dextran (FITC–DEX, *Mw* = 250000 Da, *Ex*: 488 nm/*Em*: 520 nm), propylene glycol monomethyl ether acetate (PGMEA, ≧99.5%) and isopropyl alcohol (IPA, ≧99.9%), 3‐(trimethoxysilyl)propyl methacrylate (TMSPMA) were purchased from Sigma‐Aldrich. Acetone (99.5%) and ethanol (99.5%) were purchased from Kishida Chemical Co., Ltd. (Osaka, Japan). Glass slides (S1112) were purchased from Matsunami Glass Inc., Ltd. (Osaka, Japan). Fused silica substrates (2 inches, thickness: 500 µm ± 25 µm, optical grade: JGS1) were purchased from MicroChemicals (Ulm, Germany). The IP‐S resin was purchased from Nanoscribe GmbH (Karlsruhe, Germany). Deionized water was used throughout the experiments unless otherwise mentioned. All reagents were used as provided without any further purification.

### Microfluidic Device Fabrication

The fused silica substrates were first cleaned to remove contaminants. The cleaning process began by immersing the substrates in acetone for 10 min, followed by rinsing with IPA and deionized water. The substrates were dried under a nitrogen gas stream. For surface methacrylation, cleaned fused silica substrates were treated with TMSPMA to introduce methacrylate functional groups onto the surface. The substrates were first treated with oxygen plasma using a DC sputtering system (SC‐708; Sanyu Electron, Tokyo, Japan) to activate the surface. Immediately after plasma treatment, the substrates were immersed in a solution of 2% (v/v) TMSPMA in ethanol for 30 min at room temperature. The methacrylate substrates were stored in a desiccator until use in two‐photon polymerization (2PP), a form of direct laser writing (DLW) fabrication, with a Quantum X system (Nanoscribe GmbH, Karlsruhe, Germany), which served as a nano‐precision 3D printer for microscale fabrication. In the 2PP process, 300 µL of IP‐S resin was applied onto the methacrylated fused silica substrates. The mold for the microfluidic device was fabricated using a femtosecond laser (wavelength: 780 nm) with a laser power of 115 mW and a scanning speed of 250 mm s^−1^. After fabrication, the device was developed in PGMEA for 10 min, rinsed with IPA for 1 min, and air‐dried. The PDMS prepolymer was mixed with a curing agent at a ratio of 10:1 (w/w), poured onto a 3D printed mold, degassed under vacuum to remove air bubbles, and cured at 70 °C for 2 h. The cured PDMS replica was peeled from the mold and bonded to a glass slide after treating both surfaces with oxygen plasma. The inlet and outlet ports were punched into the PDMS replica prior to bonding. The final thickness of the cured PDMS layer was ≈5 mm ± 1 mm, which was consistent across all experiments. Experiments were conducted within 6 h after the surface activation with plasma processing of microchannels.

### Experimental Setup and Image Acquisition

For the experiments shown in Figures 1 and 2, 10% (w/v) stock solutions of PEG and DEX were prepared using deionized water. The PEG and DEX stock solutions were then mixed to achieve final concentrations of 4%:4% (w/v) and 5%:5% (w/v) for the PEG/DEX mixture. For fluorescence visualization in Figure 2, FITC‐DEX was added to the 4%:4% PEG/DEX solution at a final concentration of 0.2% (w/v). For the experiments shown in Figure 4, GFP‐expressing Escherichia coli (prepared as described in Supporting Information), T4 phage DNA, anti‐mouse IgG antibody, or 200 nm polystyrene nanoparticles were individually mixed with a solution containing 4%:4% (w/v) PEG/DEX in 1 × PBS. For DNA encapsulation, 200 nm YOYO‐1 was added to the mixture as a fluorescent dye. The final concentrations of E. coli, DNA, antibody, and nanoparticles in the solutions were 5 × 10^5^ cells µL^−1^, 6.6 ng µL^−1^, 40 ng µL^−1^, and 9.5 × 10^4^ particles µL^−1^, respectively. Each PEG/DEX solution was prepared to a final volume of 500 µL. These solutions were introduced into a straight microchannel (20 µm high × 20 µm width × 30 mm length, for detail geometry in Supporting Information) with 3 mm diameter wells at each end via capillary action. Once the microchannel was filled, an equal volume of the solution was added to another well to ensure equal hydrostatic pressure and to prevent any flow within the microchannel.

The droplet generation process was observed using an inverted fluorescence microscope (TE2000; Nikon Corporation, Tokyo, Japan) equipped with an EM (electron multiplying)‐CCD camera (Hamamatsu Photonics K.K., Hamamatsu, Japan) and a confocal laser scanning microscope (FV3000; Olympus, Tokyo, Japan). Images were acquired using software supplied by the manufacturer. 3D images of the confocal microscope were reconstructed using ImageJ 1.54 g software (NIH, USA). The binodal curve shown in Figure 1c was constructed based on turbidity and microscopic observations in PEG/DEX mixtures ranging from 1% to 10% (w/v), in reference to the method of Tsumoto et al. (2015).^[^ ^35^ ^]^

### Statistical Analysis

Droplet diameters were measured using ImageJ based on fluorescence microscopy images. For each experimental condition, ≈140 droplets were analyzed. The mean, standard deviation (SD), and coefficient of variation (CV) were calculated using Microsoft Excel. No statistical hypothesis tests were performed, as the focus was on descriptive analysis of size distributions.

### Numerical Details

Numerical calculations were carried out with a Cahn–Hilliard‐type equation by modifying the Python source code available from the open‐access version provided by the Yamanaka Laboratory at the Tokyo University of Agriculture and Technology, Japan;^[^ ^50^ ^]^ for simplicity, the numerical calculation was performed with a 2D system. The numerical simulation were performed by taking into account the gradual water elimination from the polymer solution under the simplified model with the gradual progress of the phase separation by going through the bimodal line in the phase diagram.

In order to incorporate the effect of the boundary on the time development of the phase separation, the time‐dependent Dirichlet condition^[^ ^51^, ^52^ ^]^ was adapted by considering mass conservation. The discrete domain and discrete boundary are defined as Ω^*h*^ = {(*x_i_*,*z_j_*)| 1 ≤ *i* ≤ *N_x_*, 1 ≤ *j* ≤ *N_z_*} . Accordingly, the order parameter is spatially discretized as *η*_*i*,*j*_. In the modeling of microchannel experiments under a 2D approximation, the x and z directions were regarded as the long‐axis and cross‐section of the microchannel, respectively. Furthermore, the periodic boundary condition was applied in the x‐direction. With regard to the z‐direction for the boundary around the channel surface (*j* = 1, 2 and *N_z_*,*N*_*z* − 1_), the effect of the surface affinity between the PEG and DEX for the time‐stepwise calculations was included. For instance, in the solution adjacent to the surface of *j* = 1, the time‐dependent change toward the preferable value of the parameter $\eta_{i,1}^{0}$ was assumed to be a fixed value, with the simple kinetics given by $d\eta_{i,1}^{t}/dt=k\left(\eta_{i,1}^{0}-\eta_{i,1}^{t}\right)$. The kinetic constant *k* was adopted as 1 · 10^−5^s^−1^. To fulfil the mass‐conservation, the alternation of the layer with *j* = 1 was compensated by the change of that with *j* = 2.

(4) $$\eta_{i,1}^{t+\Delta t}=\eta_{i,1}^{t}+k\Delta t\left(\eta_{i,1}^{0}-\;\eta_{i,1}^{t}\right)\;$$

(5) $$\eta_{i,2}^{t+\Delta t}=\eta_{i,2}^{t}-k\Delta t\left(\eta_{i,1}^{0}-\;\eta_{i,1}^{t}\right)$$

Δ*t*  = / *T*_ime_ / *N*_t_ is the time step, *T*_ime_ is the final time, and *N*_t_ is the total number of time steps. The same time dependence was adapted for the other side of the boundary; $\eta_{i,N_{z}}^{t}$ and $\eta_{i,N_{z-1}}^{t}$. Equations (4) and (5) ($\eta_{i,1}^{0}=\eta_{i,N_{z}}^{0}=0.4$) were adopted as the boundary condition that the surface exhibits the affinity to the PEG phase.

Since the volume of the PEG phase was greater than that of the DEX phase in the experiment, the simulation was performed with perfect homogeneity for the initial value of *η*  =  0.38 on the PEG‐rich side. In the actual experiment, mechanical mixing was initially performed and then incorporated the solution into the PDMS channel. Then, the microscopic observation was started with a lag time of a few minutes. In other words, the starting time of the observation (Δ*t*  =  0 in Figures 2 and 4) corresponded to a state with a time delay of ≈10 min from the mechanical mixing of the polymer solution. Thus, in Figure 5 such time difference was set in the following manner: the observation beginning time for the channel in the experiment (Δ*t*  =  0 s) corresponds to 800 steps, or 800 s (the step interval is 1 s in the simulation), from the homogeneous initial state in the simulation, i.e., Δ*t*  =  *t* − 800 s. Figure 5 reveals the results of the time development of micro droplets in the PEG/DEX solution generated at Δ*t*  =  2,  5,  15 and 30 min corresponding to 920, 1100, 1700 and 2600 steps from the homogeneous initial state in the calculation. The grid spacing in the computation was taken to 1.0 µm. The inner diameter of the microchannel is 20 µm, which is the same as in the experiment.

In the simulation, *D*_0_  =  1.0  ×  10^−14^ m^2^ s^−1^ was assumed independent of the polymer composition, which is three orders smaller than that of the self‐diffusion of water. The universal gas constant *R*  =  8.31 J mol^−1^ K^−1^ and temperature *T*  =  295 K was used. For the calculation of Equation (2), the parameter *α*  =  3.5  ×  10^−8^ Jm^2^ mol^−1^ was tentatively adopted, so as to reproduce the experimentally observed process of segregation under a similar spatial scale and observation time. For the calculation of Equation (3), the parameters reflecting the water content change of the polymer solution: *L*_0 _ =  7300,  *a*  =  1 0000, and   *b*  =  5.0  ×  10^−3^s^−1^ was tentatively adopted.

## Conflict of Interest

The authors declare no conflict of interest.

## Author Contributions

K.H., M.S., A.S., and K.Y. conceived and designed this study. K.H. and M.S. performed the experiments and analyzed the results. M.S. performed the numerical calculations and wrote its section. K.H. and K.Y. wrote and summarized the manuscript with feedback from coauthors. All authors reviewed the manuscript.

## Supporting information



Supplemental Movie 1

Supporting Information

## Data Availability

The data that support the findings of this study are available from the corresponding author upon reasonable request.
