# Supplementary material for: A Facile Platform for One‐Step Generation of Uniform Microdroplets through Dehydration‐Driven Phase Separation in Microfluidics
Source: Small Methods. 2025 Jun 8;10(2):2500387. doi: 10.1002/smtd.202500387 (PMC12825336; doi:10.1002/smtd.202500387)
Supplement: Supplementary file 1 — Supplemental Movie 1 [file SMTD-10-2500387-s001.pdf]

Supporting Information

A Facile Platform for One-Step Generation of Uniform Microdroplets through Dehydration-Driven Phase Separation in Microfluidics

Ken Hirano, Mayu Shono, Akihisa Shioi*, and Kenichi Yoshikawa*

Contents

S1. Supporting Figures

Fig. S1

Fig. S2

Fig. S3

Fig. S4

Fig. S5

Fig. S6

S2. Rationale for the empirical exponential fit

S3. Environmental Stabilization After Droplet Formation

S4. Preparation of GFP-Expressed Escherichia coli

S5. Experimental Conditions for Movie S1

S1. Supporting Figures

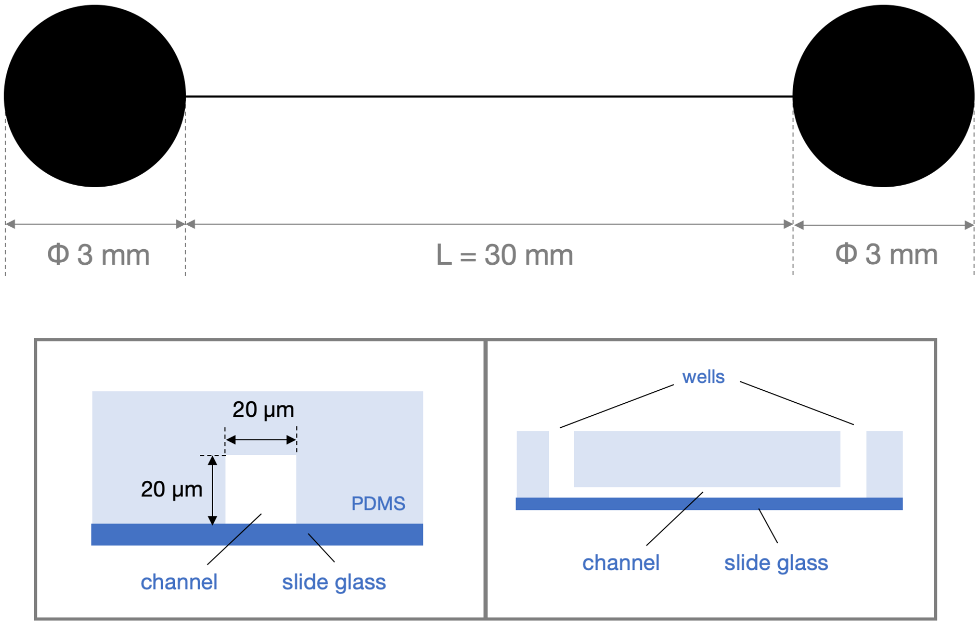

Figure S1. Geometry of the microfluidic device.
The device consists of a straight microchannel (20 μm high × 20 μm wide × 30 mm long) with 3 mm diameter wells at each end, as schematically illustrated in this figure (see also insets). The microchannel is indicated by the black line, and the wells are indicated by the black circles. A glass slide (thickness: 1.0 - 1.2 mm, size: 76 mm × 26 mm) was bonded to the bottom of the device using oxygen plasma treatment to allow for observation using an inverted fluorescence microscope.

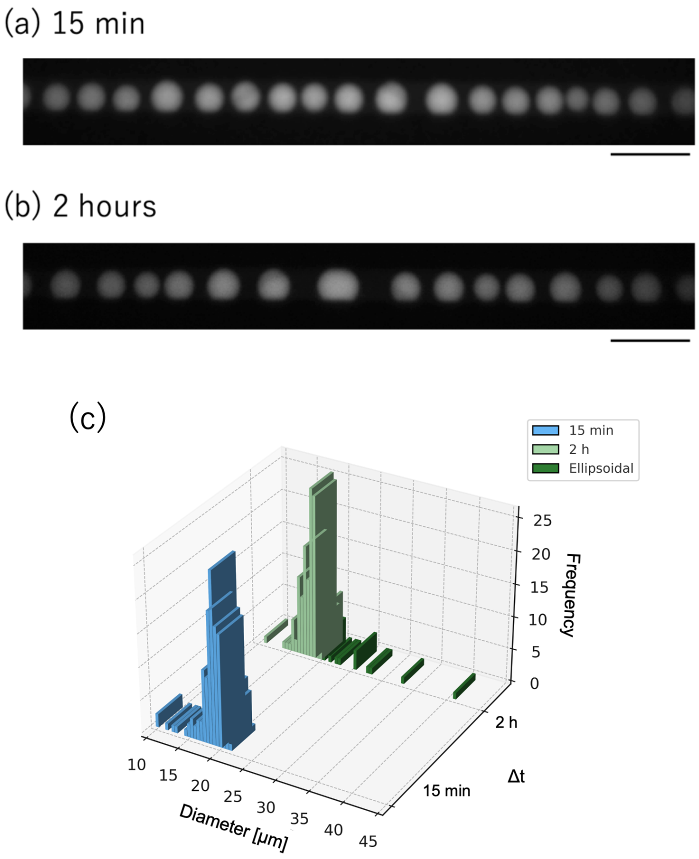

Fig. S2. Time-dependent stability of DEX-rich droplets.
(a) and (b) show fluorescence images of DEX-rich droplet array at 15 minutes and 2 hours after formation within a 20 μm PDMS microchannel, respectively. Scale bars are 50 μm. (c) Histogram of droplet diameters measured at 15 minutes (blue) and 2 hours (green), with ellipsoidal droplets are shown in red. In (c), the elapsed time points are denoted by Δt. While minor changes such as droplet coalescence or shape deformation were observed, the overall morphology and distribution remained largely stable, supporting the system's suitability for applications requiring temporal retention of compartmentalization.

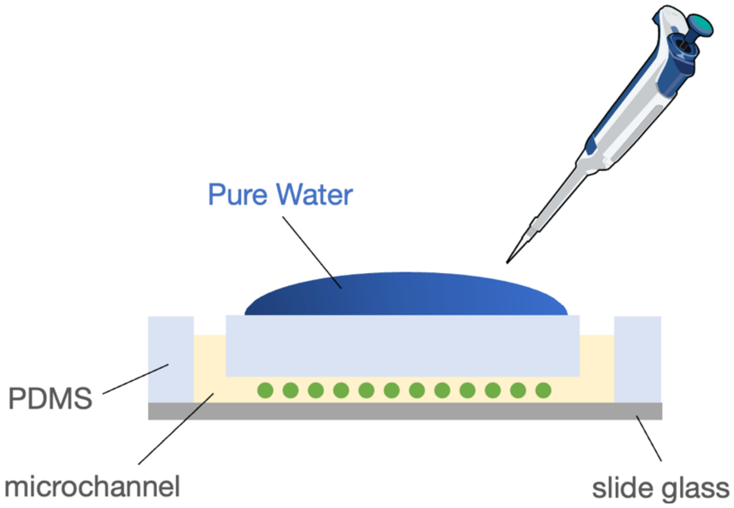

Fig. S3. Schematic illustration of the humidity stabilization strategy.
After droplet formation, a thin layer of pure water was applied over the PDMS surface to create a near-saturated local environment and suppress further dehydration. This setup enabled consistent droplet morphology during extended observation periods (see also Fig. S4 and section S3).

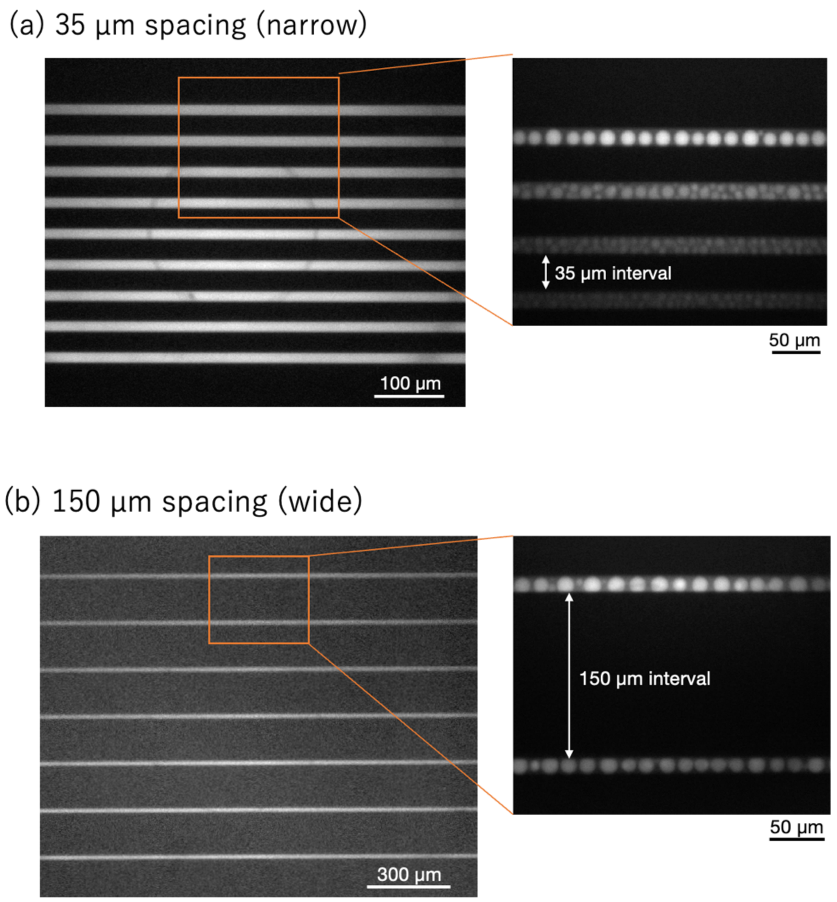

Fig. S4. Comparison of phase separation behavior in microfluidic channel arrays with different inter-channel spacing.
(a) Photograph of a densely packed PDMS microchannel array with 35 µm spacing between adjacent channels. Droplet formation occurred earlier in edge channels but was significantly delayed in the central channels, suggesting inter-channel competition for vapor diffusion. (b) Photograph of a sparsely spaced array with 150 µm inter-channel distance. All channels showed synchronized droplet formation within the same time frame, indicating that sufficient spacing mitigates diffusion interference and supports uniform phase separation.

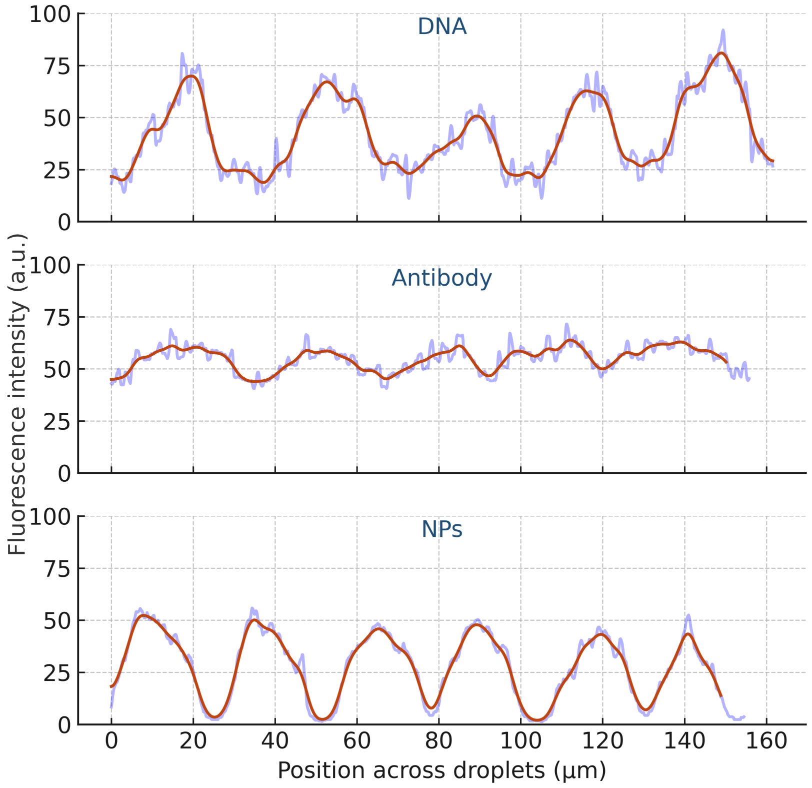

Fig. S5. Fluorescence intensity line profiles across individual dextran-rich droplets. Fluorescence signals of DNA, fluorescent nanoparticles (NPs), and fluorescently labeled antibodies were analyzed along a linear path across each droplet. Profiles were obtained using ImageJ from the corresponding fluorescence microscopy images in Figure 3. To reduce noise and improve clarity, the raw intensity profiles were smoothed using a Gaussian filter before plotting. While DNA and NPs exhibited clear localization within the droplets, the signal for antibodies was less pronounced, suggesting lower partitioning efficiency under the current buffer conditions (PBS). These profiles support the qualitative observations shown in the main figure and underscore the variability in encapsulation behavior depending on molecular properties.

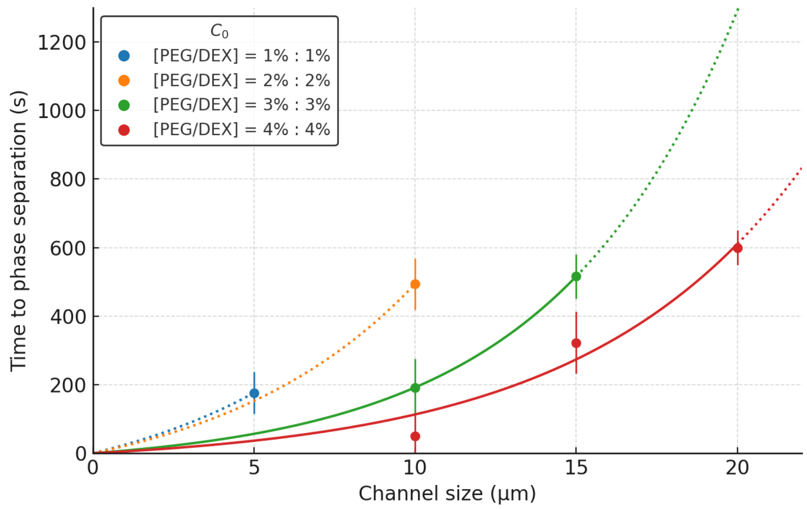

Fig. S6. Dependence of phase separation time on channel size for different initial polymer concentrations.

Solid curves represent empirical exponential fits to the experimental data, while dotted lines indicate extrapolations beyond the measured range. The results highlight that higher initial concentrations and smaller channel dimensions both promote faster phase separation, consistent with diffusion-limited dehydration behavior. Details of the fitting rationale are provided in Supporting Information Section S2.

S2. Rationale for the empirical exponential fit

1. Diffusion-limited dehydration in PDMS (Fick's 2nd law)

We consider one-dimensional diffusion of water from the PEG/DEX solution into the surrounding PDMS. The water concentration $C\ (x,t)$ in the PDMS is governed by Fick's second law:

$$\frac{\partial C}{\partial t} = D\frac{\partial^2 C}{\partial x^2},$$

where $C\ (x,t)$ is the water concentration at position x and time t in the PDMS, D is the diffusion coefficient of water in PDMS.

We assume a semi-infinite PDMS domain, initially equilibrated with the ambient humidity at concentration C_{air}, and the boundary at $x = 0$ (the solution–PDMS interface) is maintained at a fixed concentration C_{sat} for all $t > 0$.

The normalized concentration field is then defined as:

$$C^*(x,t) = \frac{C(x,t) - C_{\mathrm{air}}}{C_{\mathrm{sat}} - C_{\mathrm{air}}},$$

where $C_{\mathrm{sat}} = P_{\mathrm{sat}}/RT$, $C_{\mathrm{air}} = P_{\mathrm{air}}/RT$, and P_{sat} and P_{air} correspond to the saturation vapor pressure at the absolute temperature T and the partial pressure of H_2O in ambient air, respectively. R is the gas constant.

In this form, $C^*\ (x,t)$ is a dimensionless variable that ranges from 0 (ambient H_2O concentration) to 1 (fully saturated interface), and serves as a normalized measure of diffusion progress over time and distance.

The classical solution to Fick's second law under these conditions is given by:

$$C^*(x,t) = \ erfc\left(\frac{x}{2\sqrt{Dt}}\right),$$

where $erfc\ (\cdot)$ denotes the complementary error function:

$$erfc(z) = \frac{2}{\sqrt{\pi}}\int_z^{\infty} e^{-u^2}\,du.$$

This expression describes transient diffusion into a semi-infinite medium with a fixed boundary concentration. It is a standard analytical solution widely used in moisture uptake, solvent diffusion, and heat transfer problems involving similar initial and boundary conditions (see, e.g., Crank, The Mathematics of Diffusion, 2nd ed., Oxford University Press, 1975).
The water flux $J(t)$ at the PDMS interface ($x = 0$) is given by the gradient of the concentration profile:

$$J(t) = -D\frac{\partial C}{\partial x}\bigg|_{x=0} = \frac{\sqrt{D}(C_{\mathrm{sat}} - C_{\mathrm{air}})}{\sqrt{\pi t}},$$

where the last expression follows from differentiating the erfc solution.
The cumulative amount of water absorbed into the PDMS per unit area is then obtained by integrating the flux:

$$Q(t) = \int_0^t J(t')\,dt' = 2J_0\sqrt{t},$$

where the effective surface flux constant J_0 is defined as:

$$J_0 = \frac{\sqrt{D}(C_{\mathrm{sat}} - C_{\mathrm{air}})}{\sqrt{\pi}}.$$

This result shows that the cumulative uptake $Q(t)$ increases with the square root of time, which is characteristic of diffusion-limited mass transfer into a semi-infinite medium.

(Note: In this section, the spatial variable x is defined as the coordinate normal to the solution–PDMS interface to describe diffusion into the bulk PDMS. This differs from the longitudinal coordinate z used in the numerical phase-field simulations in the main text. The notation x is adapted here to match the conventional representation of one-dimensional diffusion.)

2. Time to reach the critical concentration $C^*_{binodal}$

We define $T(a)$ as the time required for the polymer concentration within the microchannel to increase from the initial value C_0 to the binodal threshold concentration C^*_{binodal} due to dehydration into the PDMS walls. Here, a denotes the width (or height) of a square

microchannel, which determines the surface-to-volume ratio of the system. As water is absorbed by the surrounding PDMS, the solute concentration increases until phase separation occurs.

Under the assumptions of uniform initial composition and diffusion-limited water uptake, the fractional volume loss due to dehydration is:

$$\frac{\Delta V}{V} = \frac{8J_0\sqrt{t}}{a},$$

and the corresponding volume change required to reach the binodal condition is:

$$\frac{\Delta V}{V} = 1 - \frac{C_0}{C^*_{\mathrm{binodal}}}.$$

Equating the two expressions and solving for t yields:

$$t = \left[\frac{a}{8J_0}\left(1 - \frac{C_0}{C^*_{\mathrm{binodal}}}\right)\right]^2 \qquad \text{(Eq. S1)}$$

Equation (S1) predicts a quadratic dependence $t \propto a^2$ and describes our data reasonably well (see Fig. S6).

3. Empirical determination of *A* and *k*

In the following, we use T_{exp} to represent the experimentally measured time to phase separation, and a_{exp} to denote the corresponding microchannel width used in the measurement. For the limited range a = 5–20 μm used in this study, the relation can be captured equally well by a two-parameter exponential form:

$$T(a) = A\left(e^{ka} - 1\right), \quad T = 60\,t, \qquad \text{(Eq. S2)}$$

because $e^{ka} - 1 \approx ka + (ka)^2/2 + \ldots$. A and k are fitting parameters. Truncating at the quadratic term recovers Eq. (S1), but the full exponential has two practical advantages:

- It forces the curve through the origin ($T = 0$ when $a = 0$),
- It remains monotonic and well-behaved when extrapolated to larger a.

For C_0 = 3% and 4%, we have ≥2 data points, allowing a direct least-squares fit of Eq. (S2). The fitted slopes ($k_{3\%}$, $k_{4\%}$) differ only ≈15%, so their average slope is:

$$k_{\text{avg}} = \frac{k_{3\%} + k_{4\%}}{2} = 0.11\ \mu\text{m}^{-1}.$$

For C_0 = 1% and 2%, only one point is available; thus, k_{avg} is fixed and A is obtained analytically:

$$A = \frac{T_{\text{exp}}}{e^{k_{\text{avg}} a_{\text{exp}}} - 1}.$$

4. Resulting master plot (Fig. S6)

- Solid lines: Eq. (S2) within the experimental range $0 \le a \le a_{\text{max}}$.
- Dotted lines: exponential extrapolation beyond the last data point.
- Filled circles: experimental values (all concentrations).

The RMS deviation between Eq. (S2) and the experimental times is ≤90 s, confirming that the exponential approximation is adequate for illustrating the size dependence over the practical channel widths used here. The parameter a_{max} refers to the largest channel size evaluated for each initial polymer concentration condition.

Table S1. Summary of variables and parameters used in the theoretical model.

Symbol	Description
$C(x,t)$	Water concentration in PDMS at position x and time t (mol/m^3)
D	Diffusion coefficient of water in PDMS (m^2/s)
C_{sat}	Saturated water concentration at the PDMS–air interface (mol/m^3)
C_{air}	Initial water concentration in PDMS under ambient humidity (mol/m^3)
$C^*(x,t)$	Normalized water concentration
P_{sat}	Saturation vapor pressure at the absolute temperature T (Pa)
P_{air}	Partial pressure of H_2O in ambient air (Pa)
$J(t)$	Water flux at the PDMS–solution interface at time t (mol/m^2·s)
$Q(t)$	Cumulative amount of water absorbed per unit area over time t (mol/m^2)
a	Channel width (or height, assuming square cross-section) (m)
a_{exp}	Channel size used in the experiment (μm)
a_{max}	Largest channel size evaluated for each initial polymer concentration condition (μm)
V	Volume of the PEG/DEX solution inside the microchannel (m^3)
C_0	Initial total polymer concentration in the PEG/DEX mixture (wt%)
C^*_{binodal}	Threshold polymer concentration at which phase separation occurs (wt%)
$T(a)$	Time required to reach phase separation in a channel of width a (s)
T_{exp}	Experimentally measured time to phase separation (s)
A	Fitting parameter representing the amplitude of the exponential function in Eq. (S2)
k	Fitting parameter corresponding to the rate constant in Eq. (S2) (μm^{-1})
k_{avg}	Average rate constant obtained from fitting the experimental data (s^{-1})
$k_{3\%}$, $k_{4\%}$	Fitting parameters corresponding to initial polymer concentrations of 3% and 4% (s^{-1})

S3. Environmental Stabilization After Droplet Formation

After the formation of droplets (typically about 10 minutes after introducing the PEG/DEX solution), a thin layer of deionized water (~500 µL) was applied over the PDMS surface to equalize the local humidity gradient and suppress further water loss. This step helped to stabilize the droplet environment and minimize day-to-day variation caused by ambient humidity fluctuations. All experiments were conducted at room temperature (~24° C) under the humidity of 60-90%.

S4. Preparation of GFP-Expressed Escherichia coli

To produce Escherichia coli JM109 cells that express GFP, the pAcGFP1 vector (Clontech, USA) was introduced into JM109 using Z-competent cells (Zymo Research, USA). Briefly, 100 ng of the pAcGFP1 plasmid was combined with 50 µL of Z-competent JM109 cells and gently mixed. The mixture was then chilled on ice for 10 minutes, subjected to a 42 °C heat shock for 45 seconds, and immediately returned to ice for an additional 2 minutes. Subsequently, 950 µL of SOC medium was added, and the culture was incubated at 37 °C with shaking at 200 rpm for 1 hour. The transformants were spread on LB agar plates containing 100 µg mL-1 ampicillin and grown overnight at 37 °C. Colonies that displayed green fluorescence under UV illumination were selected as positive clones.

A single GFP-positive colony was used to inoculate 5 mL of LB broth supplemented with 100 µg mL-1 ampicillin. This culture was incubated at 37 °C with shaking at 200 rpm until the OD600 reached 0.6–0.8. Cells were then harvested by centrifugation at 3,000×g for 5 minutes, and the supernatant was discarded. The resulting pellet was resuspended in 1× phosphate-buffered saline (PBS), and this washing step (centrifugation plus resuspension) was repeated twice more to completely replace the growth medium with PBS. The final cell suspension was stored at 4 °C and used on the same day for further analysis.

S5. Experimental Conditions for Movie S1

Movie S1 shows real-time observation of a 4%:4% (w/v) PEG/DEX solution introduced into a microchannel with a height and width of 20 µm. The video starts 2.5 min after the well-mixed solution is placed in one well of the chip and continues for 14 min (time-lapse images from this video are shown in Figure 2). The observation was performed using an inverted fluorescence microscope. The DEX solution contained FITC-DEX for fluorescence visualization. Due to file size limitations, the video is presented at 20× speed with reduced image quality.
